# Acute Effects of Percussive Therapy on Thigh Muscle Microcirculation and Oxygenation

**DOI:** 10.3390/jfmk11020154

**Published:** 2026-04-14

**Authors:** Vanessa Wellauer, Johannes Benrath, Rens Baeyens, Erich Hohenauer, Ron Clijsen

**Affiliations:** 1 Rehabilitation and Exercise Science Laboratory (RESlab), Department of Business Economics, Health and Social Care, University of Applied Sciences and Arts of Southern Switzerland, Weststrasse 8, 7302 Landquart, Switzerland; benrath.johannes@gmail.com (J.B.);; 2 Cosys-Lab, Faculty of Applied Engineering, University of Antwerp, Groenenborgerlaan 171, 2020 Antwerp, Belgium; rens.baeyens@uantwerpen.be; 3 Flanders Make Strategic Research Centre, Oude Diestersebaan 133, 3920 Lommel, Belgium; 4 Department of Movement and Sport Sciences, University of Fribourg, Bd de Pérolles 90, 1700 Fribourg, Switzerland; 5 Faculty of Sport Sciences, University of Poitiers, 8 All. Jean Monnet, 86000 Poitiers, France; 6 Department of Physiotherapy, International University of Applied Sciences THIM, Weststrasse 8, 7302 Landquart, Switzerland; 7 Department of Movement and Sport Sciences, Vrije Universiteit Brussel, Pleinlaan 2, 1050 Brussels, Belgium; 8 Health Department, Bern University of Applied Sciences, Murtenstrasse 10, 3008 Bern, Switzerland

**Keywords:** massage therapy, massage gun, muscle perfusion, recovery, tissue oxygenation

## Abstract

**Background**: Adequate muscle perfusion, particularly at the level of muscle microcirculation (MM), is essential for muscle function, recovery, and tissue health. Percussive therapy (PT) is increasingly used to support recovery and injury prevention and has shown consistent benefits for range of motion and perceived recovery. However, the underlying physiological mechanisms remain insufficiently understood, and evidence regarding its effects on MM is limited. This study investigated the acute effect of a single PT session on MM and muscle oxygen saturation (SmO_2_). **Methods**: Twenty-two healthy volunteers (24.2 ± 3.0 years) underwent a single PT application (two or four minutes) to the thigh using a handheld percussive device. MM, SmO_2_, and the perceived somatosensory sensation (PSS) were assessed at baseline and at five-minute intervals up to 40 min post-application. Data were analyzed using linear mixed models adjusted for age, lower-body fat percentage, and intervention duration. **Results**: A significant main effect of time was found for both MM and SmO_2_. MM increased significantly compared to baseline from 5 to 15 min post-application (all *p* < 0.001), while SmO_2_ increased immediately after PT and remained elevated throughout the 40-min observation period (all *p* < 0.001). PSS increased significantly during the first 20 min (all *p* < 0.02) before returning to baseline. **Conclusions**: A single PT application was associated with transient increases in MM and sustained elevations in SmO_2_, along with associated subjective sensations. These time-associated changes suggest that PT may enhance local muscle perfusion and therefore contribute to the understanding of its physiological mechanisms.

## 1. Introduction

Muscle tissue perfusion is essential for supporting muscle function, recovery, and injury prevention, as insufficient perfusion leads to muscle weakness, muscle cell apoptosis, and reduced regenerative capacity [1,2]. Adequate blood flow, particularly at the level of muscle microcirculation (MM), ensures the delivery of oxygen and substrates to the tissue and removal of metabolic by-products [3,4,5]. These dynamic processes are fundamental for cellular function, repair, and adaptation [6,7]. Therefore, impaired perfusion can hinder these processes and potentially prolong recovery and compromise tissue health. Enhancing tissue perfusion has long been considered a key strategy to support recovery and maintain muscle function in both athletic and clinical settings. Common approaches to post-exercise recovery include cold-water immersion, low-intensity exercise, massage, and compression [8,9,10].

Percussive therapy (PT) has gained attention as a therapeutic tool to support post-exercise recovery, injury prevention, and pre-activity preparation [11]. PT devices are handheld, self-administered tools that deliver rapid, short mechanical impulses to the targeted tissue, mainly muscle tissue. They are claimed to reduce muscle stiffness and pain, improve range of motion (ROM), and promote recovery [12,13]. Its practical advantages, portability, ease of use, and relatively low costs have contributed to its widespread acceptance and use. However, despite the growing popularity, current use is often guided more by practitioner experience than by robust mechanistic evidence, with increased local blood flow frequently assumed to be a primary underlying mechanism [11]. Previous research on PT and related mechanical interventions, such as vibration therapy, foam rolling, and massage, has predominantly focused on outcomes such as ROM and muscle stiffness [14,15,16]; perceived recovery and pain [17,18,19]; and performance measures, such as maximum strength and jumping performance [17,19,20,21]. While there is consensus regarding its beneficial effects on ROM and flexibility, findings regarding performance outcomes remain inconsistent [22]. A comparative study indicates that PT offers superior benefits to foam rolling and mechanical vibration therapy in restoring muscle compliance and reducing stiffness [23].

From a physiological perspective, these interventions are thought to act through a combination of neuronal, mechanical, and vascular mechanisms [22]. However, the vascular responses reported in the literature are often based on measurements of macrovascular blood flow (e.g., arterial flow and velocity) or cutaneous microcirculation (e.g., skin-level perfusion) [24]. Increases in arterial or cutaneous blood flow may occur without corresponding improvements in intramuscular perfusion, as these compartments are regulated differently and may respond differently to mechanical stimuli such as PT [25,26].

To date, only a small number of studies have attempted to investigate the physiological mechanisms underlying PT more directly. For example, increased popliteal arterial blood flow has been observed following PT application to the calf muscle [27], and recent work has reported increases in skin temperature and superficial microcirculation following local PT [28]. While these findings suggest a vascular response to PT, they do not provide direct insight into functionally relevant intramuscular perfusion, which is critical for muscle recovery processes. Consequently, despite the popularity of PT devices, there remains a clear gap in the literature regarding their direct effects on intramuscular microcirculation and local muscle oxygenation.

Therefore, this quasi-experimental repeated-measures study without a control condition aimed to investigate the acute effect of a single PT session applied to the thigh muscle on MM and SmO_2_. Additionally, perceived somatosensory sensation (PSS) was assessed to account for the subjective experience of the intervention. It was hypothesized that a single PT session applied to the thigh would result in acute increases in MM and SmO_2,_ and that these changes would be accompanied by corresponding changes in PSS.

## 2. Materials and Methods

### 2.1. Study Design and Participants

This study was designed as a quasi-experimental repeated-measures study without a control condition. A total of 22 healthy volunteers from a mixed university population (students) were enrolled. No formal sample size calculation was performed prior to the study. Recruitment was conducted via information screens on campus and informational flyers. Before enrollment, all participants were screened according to the inclusion and exclusion criteria. Eligible participants were required to be older than 18 years, have intact skin over the thigh, and be able to fully understand the study procedures. Exclusion criteria included musculoskeletal injuries, hypoesthesia or dysesthesia of the lower limb, metal implants in the intervention area, and the use of anticoagulants or pain medications. Pregnancy and lactation were also exclusion criteria. Due to the absence of a control condition, each participant served as their own reference across repeated measurements. Prior experience with percussive therapy was not an exclusion criterion. Participants were randomly allocated to either a 2 min or 4 min PT condition. The allocation sequence was generated by the investigator (JB) prior to data collection using a pre-balanced set of lots (11 lots per group). Participants were enrolled and screened by the same investigator (JB), who also performed the allocation by presenting the sealed lots to each participant for self-selection immediately before measurement. Due to the nature of the intervention, neither participant nor assessor blinding was feasible. The treated leg (left or right thigh) was determined by coin toss immediately thereafter. Written informed consent was obtained from all participants. All measurements were conducted between 1 March and 1 April 2024. The study was conducted in accordance with the Declaration of Helsinki and was approved by the Swiss Ethical Committee of Zurich (BASEC-No. 2023-02093). The characteristics of the participants and the distribution of sex and intervention leg are presented in Table 1.

### 2.2. Experimental Overview

The measurements were conducted in a controlled laboratory environment (21.8 ± 0.38 °C and 39.9 ± 0.99% humidity) with room temperature and humidity controlled using a multimeter (Voltcraft MT-52, Conrad Electronic SE, Hirschau, Germany). The entire experimental procedure was completed within one day. Participants were asked to refrain from strenuous physical activity and to maintain their habitual fluid intake in the 24 h prior to measurement, in accordance with standard laboratory procedures. Measurements were predominantly conducted during midday and early afternoon hours. As all analyses were based on within-subject comparisons and MM data were normalized to individual baseline values recorded on the same day, potential diurnal variation was considered unlikely to substantially influence the outcomes. Before intervention, body height (cm), body weight (kg), and estimated lower-body fat (%) were measured using a stadiometer (GPM, Zurich, Switzerland) and a digital weight scale (TANITA-TBF 611 scale, Tokyo, Japan). Following anthropometric measurements, participants were instructed to lie in a supine position for a 15 min acclimatization period, during which they were prepared for subsequent measurements (explained below). After the acclimatization period, all baseline measurements (BL) were taken. All outcome parameters were then reassessed immediately after PT (T0) and subsequently at 5 min intervals up to 40 min post-intervention (T1–T8).

### 2.3. Muscle Microcirculation

**Figure 1:**
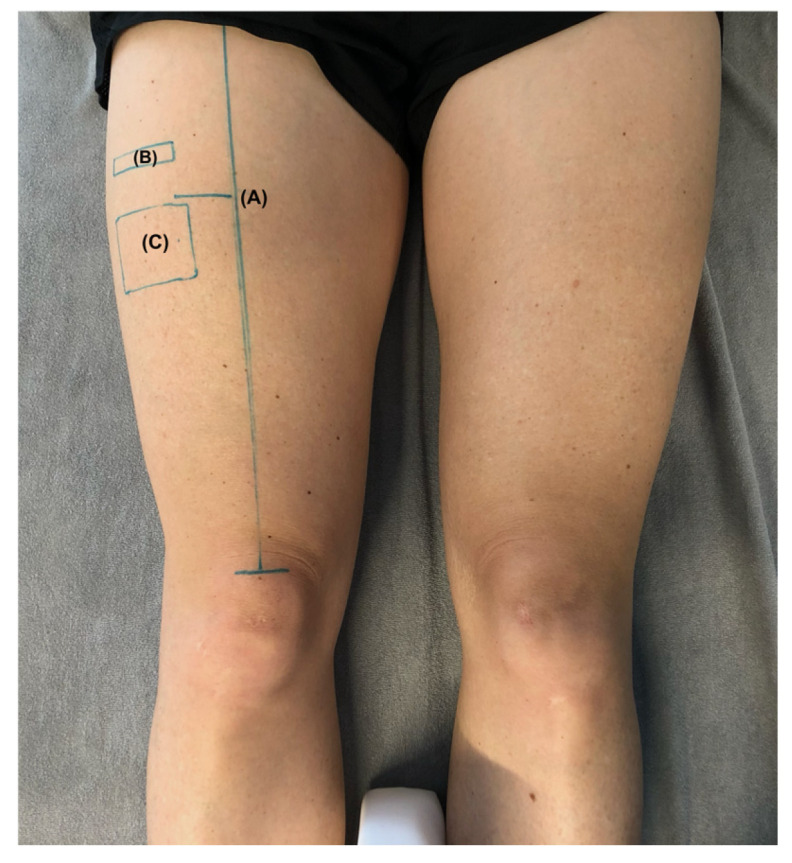
Probe placement markings for the ultrasound probe and near-infrared spectroscopy probe: (A) midpoint of the spina iliaca anterior superior and the superior border of the patella; (B) position for the ultrasound probe; (C) position for the NIRS device.

The thigh’s MM was assessed in the previously marked area using the Micro V application (MyLabTwice, Esaote, Genoa, Italy). Micro V represents an advanced evolution of Doppler technology specifically designed to overcome the limitations of conventional power Doppler in detecting microvasculature. While power Doppler ultrasound is adequate for standard vascular imaging, Micro V excels at visualizing small vessels and slow flows that would be missed by conventional Doppler techniques, making it particularly valuable for characterizing tissue perfusion and lesion vascularization [29,30]. The minimal-pressure technique was employed to assess blood flow without causing occlusion [31]. To enhance measurement reliability, participants’ legs were secured together using hook-and-loop fasteners. Measurement sites for the ultrasound probe and the near-infrared spectroscopy (NIRS) sensor were identified and marked with a skin-friendly highlighter prior to data collection. Two anatomical landmarks were palpated and marked: the spina iliaca anterior superior and the superior border of the patella. The distance between these two points was measured, and the midpoint was determined (Figure 1 (A)). The ultrasound probe was positioned 3 cm lateral and 2.5 cm proximal to the midpoint (Figure 1 (B)). For each measurement, a series of images was acquired, and a five-second video sequence was recorded. To quantify blood flow changes from Micro V images, a custom-built MATLAB (Version 2022b) program was used. The program calculates a single value score representing the intramuscular flow activity increase/decrease in a Micro V image with respect to the reference image. First, the custom color scale used in the Micro V image is discretized so that each pixel in the image can be mapped to the level corresponding to the lowest Manhattan distance between the pixel’s color and the color corresponding to that level. For example, if we use three discretization levels, each pixel will be mapped to one of the following colors, which minimizes the Manhattan distance between the r, g, and b components: dark red, orange, and dark blue. Second, a gamma transformation is performed to adjust the sensitivity to low-intensity values (i.e., red pixels). A gamma value of one was adopted during this study, resulting in a linear relationship between the perfusion intensity (i.e., the discretization level) of a pixel and its contribution in the final calculation. Third, a threshold was put in place, which nullifies the contribution of pixels below a predefined perfusion level threshold in order to mitigate the influence of background noise on the final score. Finally, all pixels’ intensities (after the gamma transformation and the thresholding) are summed to obtain the intramuscular flow activity score of a PDUS image, which is then normalized to eliminate the score’s sensitivity to the image size and resolution. For each measurement interval, the image with the maximum perfusion value was identified for each participant to be included in the statistical analysis. To support reproducibility and test-retest reliability, the image-processing pipeline incorporates three user-defined parameters (precision, gamma, and threshold), which can be saved and consistently reapplied across analyses. These parameters were made adjustable to account for the relative nature of the measurements and variability in image quality. For all comparative analyses, identical parameter settings were used across image sets to ensure methodological consistency and inter-test reliability.

Prior to statistical analysis, all MM data underwent a predefined quality control procedure, regardless of group allocation or expected intervention effects. Raw recordings were visually inspected for signal integrity and artifacts (e.g., motion artifacts, loss of probe contact, or signal saturation). Baseline measurements were required to meet minimum quality criteria, including stable signal acquisition and analyzable image contrast. Datasets were excluded if (i) baseline recordings were technically corrupted (N = 1), preventing reliable quantification, or (ii) post-intervention recordings showed clear technical artifacts (e.g., abrupt signal saturation or non-physiological spikes consistent with acquisition errors), preventing valid analysis (N = 3).

### 2.4. Muscle Oxygen Saturation

SmO_2_ was measured using a portable NIRS device (MOXY-Monitor Model 3, Fortiori Design LLC, Hutchinson, MN, USA). Oxygenated hemoglobin (oxyHb), deoxygenated hemoglobin (deoxyHb), and total hemoglobin (TotHb) were assessed in arbitrary units, and SmO_2_ was calculated as oxyHb/TotHb × 100 value (%). NIRS is a non-invasive method widely used to assess skeletal muscle oxygenation and oxidative metabolism and has demonstrated good validity compared with reference methods such as phosphorus magnetic resonance spectroscopy under controlled conditions. However, its accuracy and reliability may vary depending on factors such as local adipose tissue thickness, probe placement, and measurement protocol [32,33]. Lower-body fat percentage was included as a covariate in all statistical models to partially account for inter-individual differences in subcutaneous tissue depth. Although site-specific adipose tissue thickness at the probe location was not measured, lower-body fat percentage was considered a pragmatic proxy given the absence of a dedicated skinfold or ultrasound measurement at the measurement site. To minimize potential placement variability, the exact probe position was marked on the skin before the first measurement at 3 cm lateral and 2.5 cm distal to the same midpoint described above (Figure 1 (C)). This position mark was used as a reference for the repositioning of the sensor after PT application. The sensor was reattached without additional manual pressure and secured with self-adhesive tape. Once in place, the sensor was not touched or repositioned for the remaining follow-up measurements. Data were recorded and tracked using IDIAG MOXY-Software (Version 1.1, IDIAG AG, Rapperswil, Switzerland).

### 2.5. Somatosensory Sensation Ratings

Participants’ perceived somatosensory sensation (PSS) of the intervention area was assessed using a five-point Likert scale. Participants rated their agreement with the statement “I feel a tingling or pulsing sensation in the area of intervention” (1 = “do not agree at all”, 5 = “totally agree”). This approach is based on previous studies evaluating somatosensory qualities such as tingling, pulsing, and warmth after manual or percussive interventions [34,35,36]. The PSS provides a quantitative measure of acute changes in subjective somatosensory perception of the intervention area.

### 2.6. Percussive Therapy

Upon completion of the acclimatization period and BL measurements, PT intervention was administered to the ventrolateral thigh using a handheld percussive massage device (Theragun 3G Pro, Therabody, Los Angeles, CA, USA) with the “large ball” attachment. The device was applied using only its own weight without any additional manual pressure, at a fixed frequency of 2400 percussions/min and an amplitude of 16 mm, and maintained in a perpendicular orientation to the tissue throughout the intervention. To ensure consistency, all PT applications were carried out by the same trained examiner (JB) following a standardized protocol. The device was moved across the ventrolateral thigh in a predefined pattern, comprising one minute of horizontal movement (alongside the vastus lateralis muscle) followed by one minute of vertical movement (perpendicular to the muscle fibers). For the 4 min condition, this sequence was repeated once. Although efforts were made to standardize the application, minor operator-dependent variations in movement speed and dwell time cannot be completely ruled out.

### 2.7. Statistical Analysis

The demographic and anthropometric data of all participants are presented in Table 1. For each outcome variable (MM, SmO_2_, PSS), linear mixed-effects models (LMM) were fitted using restricted maximum likelihood estimation. A random intercept for each subject was included to account for repeated measures and individual baseline differences. Time was modeled as a categorical fixed effect with 10 levels (BL, T0–T8), using treatment contrasts with BL as the reference category. Random slopes for time were considered but omitted, as estimating random slopes for a 10-level categorical time factor across 22 subjects (18 for MM) would require as many random effects parameters (N = 220) as observations, rendering the model non-identifiable.

All models were adjusted for lower body fat percentage, age, and intervention duration (2 min and 4 min) to control for their potential influence on outcomes. Model assumptions were evaluated by visual inspection of residual plots and Q-Q plots. Residual diagnostics indicated approximate normality for SmO_2_ and PSS. For MM, heteroscedasticity was observed and attributed to between-subject differences in absolute MM magnitude propagating through baseline normalization (BL = 100%). Given the robustness of LMM fixed effect estimates to moderate violations, the model was retained on the normalized scale. PSS was measured on an ordinal Likert scale but analyzed parametrically, which is commonly considered acceptable when Likert scales have multiple categories and approximate interval-level measurements, with parametric models shown to be robust to such violations [37,38]. This approach also ensured analytical consistency across outcomes.

MM data were normalized with baseline values set as 100% to quantify relative temporal changes. Four participants were excluded from MM analysis due to technically invalid measurements, as described in Section 2.3, resulting in a final sample of N = 18 (2 min subgroup: N = 8; 4 min subgroup: N = 10). All remaining datasets were complete with no missing observations. A sensitivity analysis including the three participants excluded due to non-physiological signal artifacts is reported in Supplementary Table S4. The direction of all effects remained consistent with the primary analysis; however, statistical significance was not maintained, attributable to the substantially increased residual variance introduced by the artifact-affected recordings.

Pairwise comparisons of estimated marginal means were performed to compare each post-intervention time point (T0–T8) with BL, with Bonferroni correction applied for multiple testing. Effect sizes (Cohen’s d) were calculated as the ratio of the estimated marginal mean difference to the residual standard deviation of the respective LMM (residual-based Cohen’s d). As this approach is based solely on residual variance and does not incorporate between-subject variance components, these values may not be directly comparable to Cohen’s d derived from traditional ANOVA or t-test approaches and should therefore be interpreted accordingly.

The subgroup analyses for the 2 min and 4 min intervention groups were conducted as separate exploratory LMMs, each examining time effects within the respective group while adjusting for lower body fat (%) and age. Post hoc pairwise comparisons were conducted using Bonferroni correction. As intervention duration was included as a covariate in the primary model rather than as a between-group factor, a formal time x intervention duration interaction could not be estimated within the present design. These subgroup analyses are therefore purely descriptive and hypothesis-generating, and any apparent differences in response patterns between groups should not be interpreted as evidence of statistically supported differential effects.

Statistical analysis was conducted, and figures were created using R (Version 4.3.3, R Core Team, Vienna, Austria), with the significance level set at *p* < 0.05.

## 3. Results

All descriptive statistics on raw data for MM, SmO_2_, and PSS for the overall sample and separated by 2- and 4-min intervention duration are presented in Supplementary Table S1.

### 3.1. Changes in Muscular Microcirculation

**Figure 2:**
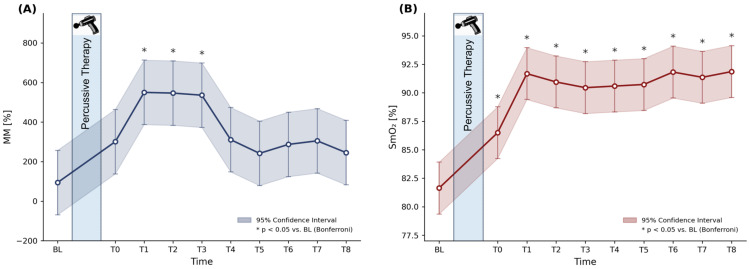
Estimated marginal means of (**A**) muscle microcirculation (MM, N = 18) and (**B**) muscle oxygen saturation (SmO_2_, N = 22) across 10 measurement time points. The shaded area represents the 95% confidence interval. * *p* < 0.05 vs. BL, Bonferroni-corrected. BL = baseline; T0 = immediately post-PT; T1–T8 = 5 to 40 min post-PT in 5 min intervals.

In the overall sample, the LMM revealed a significant main effect of time on MM (F(9,153) = 4.281, *p* < 0.001). Bonferroni-adjusted pairwise comparison showed significantly higher MM values compared to BL at T1–T3 (T1: Δ456%, 95% CI [162, 750], *p* < 0.001; T2: Δ452%, 95% CI [158, 746], *p* < 0.001; T3: Δ442%, 95% CI [148, 736], *p* < 0.001) (Figure 2A). Detailed *p*-values, confidence intervals for all estimated mean differences, and model-based effect sizes are reported in Table 2.

In the subgroup receiving a 2 min intervention, the LMM indicated a statistical trend for a time effect that did not reach significance (F(9,63) = 1.736, *p* = 0.099). Bonferroni-corrected pairwise comparisons revealed no significant differences between BL and any post-intervention time point (all *p* > 0.05). The estimated marginal means increased by Δ76 to Δ339% from BL to post-intervention, with considerable variability across measurements. In the 4 min subgroup, the LMM showed a significant main effect of time (F(9,81) = 5.007, *p* < 0.001). Pairwise comparisons revealed significantly higher MM values compared to BL at T1–T3 with large effect sizes (T1: *p* < 0.001, Cohen’s d: 1.85; T2: *p* = 0.003, Cohen’s d: 1.69, and T3: *p* = 0.002, Cohen’s d: 1.72). The corresponding estimated mean differences ranged from Δ561% to Δ612%. Detailed *p*-values and confidence intervals for estimated mean differences in the subgroups are presented in Supplementary Tables S2 and S3.

### 3.2. Changes in Muscle Oxygen Saturation

In the overall sample, the LMM revealed a significant main effect of time on SmO_2_ (F(9,189) = 18.513, *p* < 0.001). Pairwise comparison against BL confirmed significantly higher SmO_2_ values from T0 to T8 (all Bonferroni-adjusted *p* < 0.001). The mean increase compared to BL ranged from 4.9 ± 3.0% at T0 to 10.2 ± 3.0% at T8 (Figure 2B). Detailed *p*-values, estimated mean differences, confidence intervals, and model-based effect sizes are provided in Table 2.

In the subgroup receiving a 2 min intervention, SmO_2_ also showed a significant main effect of time (F(9,90) = 11.457, *p* < 0.001). Bonferroni-corrected pairwise comparisons indicated significantly higher values compared to BL for T1–T8 (all *p* < 0.001), with mean increases ranging from +9.9 ± 4.9% (T2) to +13.1 ± 4.9% (T8) and effect sizes (Cohen’s d) ranging from 2.46 to 3.26. In the subgroup receiving a 4 min intervention, the LMM likewise revealed a significant main effect of time (F(9,90) = 9.789, *p* < 0.001). Pairwise comparisons showed significantly higher SmO_2_ values compared to BL at all post-intervention time points (T0–T8: all *p* < 0.001). Mean increases ranged from +4.9 ± 3.3% (T0) to +9.2 ± 3.3% (T1), with effect sizes (Cohen’s d) ranging from 1.79 to 3.34.

### 3.3. Changes in Perceived Somatosensory Sensation

**Figure 3:**
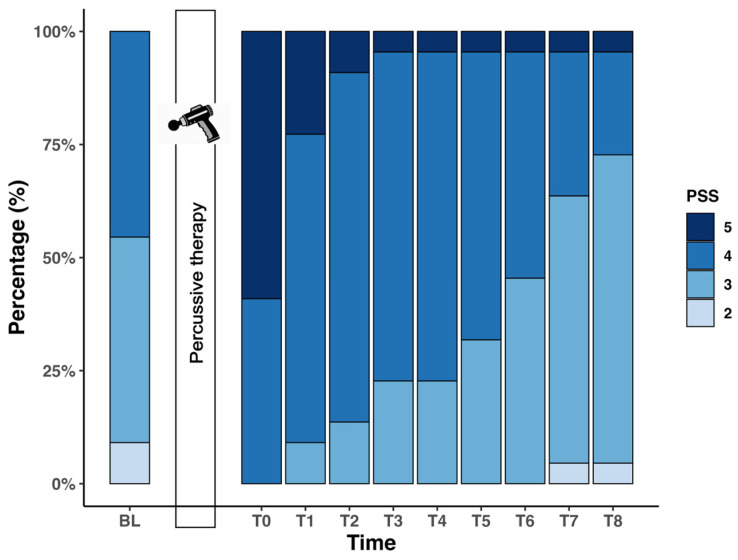
Stacked bar plot showing the percentage distribution of perceived somatosensory sensation (PSS) ratings across 10 measurement time points. N = 22 at each time point. PSS was rated on a 5-point Likert scale in response to the statement “I feel a tingling or pulsing sensation in the area of intervention” (1 = do not agree at all, 5 = totally agree).

In the overall sample, the LMM revealed a significant main effect of time on PSS (F(9,189) = 16.869, *p* < 0.001). Bonferroni-corrected pairwise comparisons showed significantly higher PSS scores compared to BL at T0–T4 (T0–T2: *p* < 0.001; T3 and T4: *p* = 0.01). The peak score of PSS was reached at T0, with an estimated mean difference of 1.2 ± 0.4 points (Figure 3). Detailed *p*-values, estimated mean differences, confidence intervals, and model-based effect sizes are provided in Table 2.

For the 2 min subgroup, LMM revealed a significant main effect of time on PSS (F(9,90) = 5.983, *p* < 0.001). Post hoc comparisons showed significantly higher scores at T0 (*p* < 0.001, Cohen’s d: 2.17) and T1 (*p* = 0.003, Cohen’s d: 1.58) compared to BL. For the 4 min subgroup, LMM likewise showed a significant main effect of time (F(9,90) = 12.345, *p* < 0.001). Pairwise comparison indicated significantly higher PSS scores compared to BL at T0–T2 (T0: *p* < 0.001, Cohen’s d: 3.21; T1: *p* < 0.001, Cohen’s d: 1.80; T2: *p* < 0.001, Cohen’s d: 1.80).

## 4. Discussion

This study investigated the acute effects of a single PT application using a handheld percussive massage device on the local perfusion of the ventrolateral thigh in healthy subjects. In line with our hypothesis, both objective perfusion parameters, MM and SmO_2_, increased significantly immediately following PT application and remained elevated up to 40 min post-application.

Following PT, MM increased significantly, peaking at five minutes post-intervention (T1) and remaining elevated up to 15 min (T3). To our knowledge, this is the first study to investigate local muscle perfusion following PT. A recent study examined the effects of a handheld percussive massage device applied to the calf muscle on popliteal artery blood flow [27]. In line with our results, they showed a significant increase in blood volume flow and mean velocity, which peaked at 1–3 min after application, depending on the application duration and frequency [27]. In our study, MM peaked around five to ten minutes after application with a mean peak increase of 456% (at T1). The delayed peak compared to Needs et al. may reflect differences in measurement location (popliteal artery vs. quadriceps muscle) and application method (proximal-distal vs. horizontally and vertically) rather than treatment duration alone. Therefore, comparisons must be made with caution: Local arterial blood flow does not necessarily reflect microvascular perfusion within the muscle tissue [39,40]. Additionally, the 5 min measurement interval in the present study does not allow for the determination of the exact peak timing. Our results are broadly consistent with previous studies using similar mechanical modalities, such as whole-body vibration (WBV) [39,41,42] and foam rolling (FR) [43], which reported transient increases in muscle blood flow lasting up to 30 min post-intervention. Nevertheless, not all studies report such effects. A recent study found no changes in microvascular blood flow in the vastus intermedius and lateralis muscles after FR [40]. Methodological differences, such as applied pressure, vibration frequency, treatment duration, and measurement site, likely play a critical role in the hemodynamic response, as is also reflected in the SmO_2_ results.

An even more pronounced and sustained response was observed in SmO_2_, which increased immediately after PT, peaked within five minutes (91.7 ± 2.3%), and remained substantially elevated throughout the 40 min observation period (Figure 2B). The consistently large model-based effect sizes (Cohen’s d = 1.3–2.8) suggest that these changes exceed what would typically be considered a small or negligible effect. This indicates that the observed responses may carry physiological relevance beyond statistical significance alone. These results align with recent findings from our laboratory, showing similar improvements in SmO_2_ after a 4 min Theragun^TM^ application in healthy female participants [28]. However, NIRS-derived measurements are sensitive to probe positioning, local tissue characteristics, and adipose tissue thickness. Although care was taken to standardize probe placement using anatomical landmarks and skin markings, the temporary removal and repositioning of the sensor during PT may have introduced measurement variability in the signal acquisition. Furthermore, lower-body fat percentage was used as a proxy for local adipose tissue, which does not fully capture site-specific tissue thickness. While these factors are unlikely to fully explain the magnitude and consistency of the observed SmO_2_ elevation, they should be considered when interpreting the sustained post-intervention response.

Previous studies examining SmO_2_ responses to comparable interventions yielded inconsistent results. A meta-analysis on WBV concluded that, overall and regardless of frequency, no alterations in SmO_2_ can be expected, despite increased peripheral blood flow [41]. In contrast, FR studies, vibrating [44] and non-vibrating [44,45], reported an immediate rise in SmO_2_ following local application. Percival et al. further observed an improved resaturation rate after passive vibrating FR, suggesting enhanced microvascular function [46]. These discrepancies likely stem from small differences in the mechanical stimulus between PT, FR, and WBV. For example, active FR may exert excessive pressure on the underlying tissue and vessels, thereby temporarily reducing perfusion [46,47]. Conversely, WBV consistently increases peripheral (particularly cutaneous) blood flow, whereas the effect on muscle oxygenation measured in deeper tissue layers is small or inconsistent. Combined with the known attenuation of vibration as it propagates through the body, WBV can be considered a relatively global stimulus. It may therefore be less effective for specifically increasing perfusion in deeper muscle layers than more localized modalities [41,48,49].

In the present study, PT was applied with minimal pressure and precise guidance of the ball attachment, which may have limited reflex muscle contraction and thus reduced vascular resistance. Moreover, alternating longitudinal and transverse application directions may have increased local shear stress. This is known to stimulate endothelial nitric oxide release and subsequent vasodilation [50,51]. These mechanisms represent plausible hypothetical explanations for the observed rise in both SmO_2_ and MM, reflecting enhanced muscle perfusion and oxygen availability. However, they were not directly measured in the present study. Beyond endothelial mechanisms, the mechanical impulses generated by PT may also promote capillary recruitment through transient modulation of arteriolar and precapillary sphincter tone, thereby increasing the number of perfused capillaries and improving local oxygen delivery [52]. A larger functional capillary surface area would lead to more efficient oxygen exchange between the blood and muscle tissue [53]. This could contribute to the consistently high SmO_2_ level during the follow-up period. Furthermore, studies have reported reduced muscle stiffness following PT [54,55], FR [56,57,58], WBV [59]. This reduction could indirectly help to facilitate perfusion by lowering intramuscular pressure. However, these mechanisms go beyond what was directly measured in the current study and should therefore be considered.

The earlier decline in MM compared to SmO_2_ during follow-up measurements may be explained by the immediate blood flow response to mechanical stimulation. This response likely returns to normal once the immediate shear and pressure stimuli diminish. In contrast, SmO_2_, measured via NIRS, is widely interpreted as an index of the balance between oxygen supply (availability) and utilization in skeletal muscle [60]. In several exercise and recovery protocols, SmO_2_ values have been observed to increase (or remain elevated) during the recovery phase, likely reflecting a state of overshoot in microvascular perfusion relative to utilization [61,62]. Due to the passive nature and the low-pressure application method in our study, oxygen demand in the muscle most likely did not increase during the observation period.

In addition to the two objective perfusion measures, PSS was assessed as an exploratory outcome to capture the subjective perception. PSS increased significantly immediately after PT and remained elevated during the early post-intervention phase, before gradually returning toward baseline. As PSS was assessed using a single, non-validated Likert-scale item, its psychometric properties are unknown. Furthermore, the unblinded study design makes these findings susceptible to expectancy effects. They should therefore be interpreted as exploratory rather than on equal footing with the objective outcomes. Nevertheless, the transient rise in PSS during the first 20 min broadly mirrors the objective MM response. It may reflect rapid activation of cutaneous mechanoreceptors and superficial vascular responses, consistent with prior reports on increased erythrocyte speed, flux, and skin temperature immediately following PT [28]. Documenting such subjective responses may still be of practical relevance, as perceived sensations can influence treatment acceptance, perceived recovery, and user adherence in applied settings.

### Study Limitations

Several limitations of this study should be acknowledged. First and foremost, the absence of a control condition prevents causal conclusions from being drawn, and all observed changes should be interpreted as time-associated responses. Second, the exclusion of four participants (18%) from MM analysis due to technically invalid measurements may have influenced the MM results. Although exclusions followed pre-specified quality-control criteria applied blind to expected effects, this relatively high exclusion rate in a small sample may have influenced the MM results. Therefore, conclusions from the MM analysis should be interpreted with particular caution. Third, NIRS-derived SmO_2_ measurements are sensitive to site-specific adipose tissue thickness, which was not directly assessed. Lower-body fat percentage was included as a proxy but may not fully reflect local tissue characteristics. Finally, the relatively small sample of 22 participants, combined with mixed-sex composition and two different application durations, limits the ability to draw sex-specific or duration-specific conclusions. Sex-related differences in subcutaneous fat thickness and hormonal profiles may influence physiological response to local PT applications and could therefore have affected perfusion outcomes [63,64]. Notwithstanding these limitations, this study provides valuable initial insights into the acute microcirculatory response to PT and offers a methodological foundation for future controlled studies.

## 5. Conclusions

A single PT application was associated with significant increases in both MM and SmO_2_ in the thigh of healthy adults, suggesting that PT may enhance local muscle perfusion. While MM returned to baseline after 15 min, SmO_2_ remained elevated for at least 40 min. These findings support the use of PT as a potential modality for enhancing passive muscle perfusion. However, due to the non-controlled design, these findings should be interpreted as exploratory, and causal conclusions cannot be drawn. Future studies with larger cohorts and an appropriate control condition should further investigate the optimal sex-specific application duration, with respect to a specific targeted muscle group, to further establish the potential of PT.

## Figures and Tables

**Table 1:** Demographic data of all participants.

Parameters	All Participants (N = 22)	2-min PT (N = 11)	4-min PT (N = 11)
Age, years	24.2 (3.0)	23.7 (2.1)	24.6 (3.8)
Body height, cm	169.7 (8.3)	170.1 (8.0)	169.3 (9.0)
Body weight, kg	63.6 (10.5)	65.1 (8.0)	62.1 (12.8)
Lower body fat, %	25.3 (8.7)	28.6 (8.7)	22.0 (7.7)
Sex, N (%)			
Male	7 (31.8)	3 (27.3)	4 (36.4)
Female	15 (68.2)	8 (72.7)	7 (63.6)
Treated leg, N (%)			
Left	15 (68.2)	9 (81.8)	6 (54.5)
Right	7 (31.8)	2 (18.2)	5 (45.5)

Data are presented as mean (SD) unless otherwise indicated. PT = percussive therapy.

**Table 2:** Mean differences by time points for muscle microcirculation, muscle oxygen saturation, and perceived somatosensory sensation in the overall dataset. Illustrated are all comparisons to BL.

Comparison	Muscle Microcirculation [%] (N = 18)			Muscle Oxygen Saturation [%] (N = 22)			Perceived Somatosensory Sensation (N = 22)		
	Difference (95% CI)	*p*-Value	Cohen’s d	Difference (95% CI)	*p*-Value	Cohen’s d	Difference (95% CI)	*p*-Value	Cohen’s d
T0–BL	206.7	0.448	0.66	4.9	**<0.001**	1.37	1.2	**<0.001**	2.67
	(−87.3, 500.7)			(1.9, 7.9)			(0.8, 1.6)		
T1–BL	455.8	**<0.001**	1.45	10.1	**<0.001**	2.84	0.8	**<0.001**	1.68
	(161.8, 749.8)			(7.1, 13.0)			(0.4, 1.6)		
T2–BL	452.3	**<0.001**	1.44	9.3	**<0.001**	2.63	0.6	**<0.001**	1.29
	(158.4, 746.3)			(6.3, 12.3)			(0.2, 1.0)		
T3–BL	441.7	**<0.001**	1.41	8.8	**<0.001**	2.49	0.5	**0.011**	0.99
	(147.7, 735.6)			(5.8, 11.8)			(0.1, 0.8)		
T4–BL	216.7	0.358	0.69	9.0	**<0.001**	2.53	0.5	**0.011**	0.99
	(−77.2, 510.7)			(6.0, 12.0)			(0.1, 0.8)		
T5–BL	147.7	1.000	0.47	9.1	**<0.001**	2.57	0.4	0.085	0.79
	(−146.3, 441.6)			(6.1, 12.1)			(−0.0, 0.8)		
T6–BL	192.9	0.602	0.62	10.2	**<0.001**	2.87	0.2	0.924	0.49
	(−101.1, 486.8)			(7.2, 13.2)			(−0.2, 0.6)		
T7–BL	210.9	0.408	0.67	9.7	**<0.001**	2.75	0.0	1.000	0.00
	(−83.0, 504.9)			(6.7, 12.7)			(−0.4, 0.4)		
T8–BL	151.4	1.000	0.48	10.2	**<0.001**	2.89	−0.1	1.000	−0.20
	(−142.5, 445.4)			(7.2, 13.2)			(−0.5, 0.3)		

Adjusted for age, lower body fat (%), and intervention duration; adjusted for multiple comparisons of marginal means using Bonferroni’s method; values marked in bold indicate statistically significant values (*p* < 0.05).

## Data Availability

Data is contained within the article or Supplementary Materials (File S1). Further inquiries can be directed to the corresponding authors.

## Supplementary Materials

The following supporting information can be downloaded at: https://www.mdpi.com/article/10.3390/jfmk11020154/s1, Table S1: Mean values (±SD) of MM, SmO_2_, and PSS by timepoints; Table S2: Mean differences by time points for MM, SmO_2_, and PSS in the 2 min intervention dataset; Table S3: Mean differences by time points for MM, SmO_2_, and PSS in the 4 min intervention dataset; Table S4: Results of sensitivity analysis; File S1: Dataset.

## Abbreviations

The following abbreviations are used in this manuscript:

PT	Percussive therapy
MM	Muscle microcirculation
SmO_2_	Muscle oxygen saturation
PSS	Perceived somatosensory sensation
BL	Baseline
ROM	Range of motion
NIRS	Near-infrared spectroscopy
LMM	Linear mixed model
WBV	Whole-body vibration
FR	Foam rolling
