# Supplementary material for: Acute Effects of Percussive Therapy on Thigh Muscle Microcirculation and Oxygenation
Source: J Funct Morphol Kinesiol. 2026 Apr 14;11(2):154. doi: 10.3390/jfmk11020154 (PMC13108165; doi:10.3390/jfmk11020154)
Supplement: Supplementary file 1 [file jfmk-11-00154-s001.zip › Table S1.pdf]

Table S1. Descriptive statistics (mean ± SD) of the muscle microcirculation [AU], muscle oxygen saturation [%], and perceived somatosensory sensation by time points (Baseline, T0 – T8) for the overall sample and the 2- and 4-minute subgroups.

	N	Baseline	T0	T1	T2	T3	T4	T5	T6	T7	T8
Muscle Microcirculation											
Overall	18	100 (0)	307 (290)	556 (495)	552 (545)	542 (448)	317 (252)	248 (168)	293 (407)	311 (255)	251 (239)
2min	8	100 (0)	176 (142)	360 (196)	416 (306)	380 (281)	229 (144)	283 (193)	439 (588)	438 (268)	361 (303)
4min	10	100 (0)	411 (340)	712 (609)	661 (677)	671 (526)	387 (302)	220 (150)	176 (98)	209 (203)	164 131)
Muscle Oxygen Saturation											
Overall	22	81.64 (10.23)	86.50 (8.36)	91.68 (5.03)	90.95 (4.99)	90.45 (4.81)	90.59 (4.40)	90.73 (4.96)	91.82 (4.41)	91.36 (4.69)	91.86 (4.63)
2min	11	80.27 (11.98)	85.09 (9.84)	91.18 (5.36)	90.18 (5.56)	90.55 (4.97)	91.36 (3.80)	91.73 (4.58)	93.00 (3.46)	92.45 (4.16)	93.36 (3.35)
4min	11	83.00 (8.50)	87.91 (6.74)	92.18 (4.87)	91.73 (4.47)	90.36 (4.88)	89.82 (5.00)	89.73 (5.33)	90.64 (5.08)	90.27 (5.12)	90.36 (5.37)
Perceived Somatosensory Sensation											
Overall	22	3.4 0.7	4.6 (0.5)	4.1 (0.6)	4.0 (0.5)	3.8 (0.5)	3.8 (0.5)	3.7 (0.6)	3.6 (0.6)	3.4 (0.7)	3.3 (0.6)
2min	11	3.5 (0.7)	4.5 (0.5)	4.2 (0.6)	3.8 (0.4)	3.9 80.5)	3.9 (0.5)	3.9 (0.5)	3.7 (0.6)	3.5 (0.8)	3.4 (0.8)
4min	11	3.3 (0.6)	4.8 (0.5)	4.2 (0.5)	4.2 (0.5)	3.8 (0.5)	3.8 (0.5)	3.5 (0.5)	3.4 (0.5)	3.2 (0.5)	3.1 (0.4)

*overall ≙ all participants; 2 minutes ≙ two-minute intervention group; 4min ≙ four-minute intervention group
