# Supplementary material for: Acute Effects of Percussive Therapy on Thigh Muscle Microcirculation and Oxygenation
Source: J Funct Morphol Kinesiol. 2026 Apr 14;11(2):154. doi: 10.3390/jfmk11020154 (PMC13108165; doi:10.3390/jfmk11020154)
Supplement: Supplementary file 1 [file jfmk-11-00154-s001.zip › Table S2.pdf]

Table S2. Mean differences by time points for muscle microcirculation, muscle oxygen saturation, and perceived somatosensory sensation in the **2-minute** intervention dataset. Illustrated are all comparisons to BL**.**

**Comparison**	**Muscle microcirculation [AU] (N=8)**			**Muscle oxygen saturation [%] (N=11)**			**Perceived somatosensory sensation (N=11)**		
	**Difference (95% CI)**	***P*-value**	**Cohen's d (95% CI)**	**Difference (95% CI)**	***P*-value**	**Cohen's d (95% CI)**	**Difference (95% CI)**	***P*-value**	**Cohen's d (95% CI)**
T0 - BL	75.9 (-284, 436)	1.000	0.30 (-0.70, 1.30)	4.82 (-0.05, 9.69)	0.055	1.20 (0.35, 2.05)	1.0 (0.44, 1.56)	**< 0.001**	2.17 (1.32, 3.02)
T1 - BL	259.9 (-100, 620)	0.381	1.04 (0.04, 2.04)	10.91 (6.04, 15.78)	**< 0.001**	2.71 (1.87, 3.56)	0.73 (0.17, 1.29)	**0.003**	1.58 (0.73, 2.42)
T2 - BL	316.4 (-44, 677)	0.128	1.26 (0.26, 2.26)	9.91 (5.04, 14.78)	**< 0.001**	2.46 (1.62, 3.31)	0.36 (-0.20, 0.92)	0.610	0.79 (-0.06, 1.64)
T3 - BL	279.8 (-80, 640)	0.263	1.12 (0.12, 2.11)	10.27 (5.40, 15.14)	**< 0.001**	2.55 (1.71, 3.40)	0.45 (-0.10,1.01)	0.208	0.99 (0.14, 1.83)
T4 – BL	129.3 (-231, 490)	1.000	0.52 (-0.48, 1.51)	11.09 (6.22, 15.96)	**< 0.001**	2.76 (1.91, 3.61)	0.45 (-0.10, 1.01)	0.208	0.99 (0.14, 1.83)
T5 - BL	182.8 (-178, 543)	1.000	0.73 (-0.27, 1.73)	11.45 (6.58, 16.33)	**< 0.001**	2.85 (2.00, 3.70)	0.45 (-0.10, 1.01)	0.208	0.99 (0.14, 1.83)
T6 - BL	338.6 (-22, 699)	0.080	1.35 (0.35, 2.35)	12.73 (7.86, 17.60)	**< 0.001**	3.17 (2.32, 4.01)	0.27 (-0.29, 0.83)	1.00	0.59 -0.26, 1.44)
T7 - BL	338.5 (-22, 699)	0.080	1.35 (0.35, 2.35)	12.18 (7.31, 17.05)	**< 0.001**	3.03 (2.18, 3.88)	0.00 (-0.56, 0.56)	1.00	0.00 (-0.85, 0.85)
T8 - BL	260.7 (-100, 621)	0.376	1.04 (0.04, 2.04)	13.09 (8.22, 17.96)	**< 0.001**	3.26 (2.41, 4.10)	0.09 (-0.65, 0.47)	1.00	0.20 (-1.44, 0.26)

*Adjusted for age and lower body fat (%); adjusted for multiple comparisons of marginal means using Bonferroni's method; values marked in bold indicate statistically significant results ($p < 0.05$).
