# Supplementary material for: Acute Effects of Percussive Therapy on Thigh Muscle Microcirculation and Oxygenation
Source: J Funct Morphol Kinesiol. 2026 Apr 14;11(2):154. doi: 10.3390/jfmk11020154 (PMC13108165; doi:10.3390/jfmk11020154)
Supplement: Supplementary file 1 [file jfmk-11-00154-s001.zip › Table S3.pdf]

Table S3. Mean differences by time points for muscle microcirculation, muscle oxygen saturation, and perceived somatosensory sensation in the **4-minute** intervention dataset. Illustrated are all comparisons to BL.

**Comparison**	**Muscle microcirculation [AU] (N=10)**			**Muscle oxygen saturation [%] (N=11)**			**Perceived somatosensory sensation (N=11)**		
	**Difference (95% CI)**	***P*-value**	**Cohen's d (95% CI)**	**Difference (95% CI)**	***P*-value**	**Cohen's d (95% CI)**	**Difference (95% CI)**	***P*-value**	**Cohen's d (95% CI)**
T0 - BL	311.3 (-111, 734)	0.351	0.94 (0.04, 1.83)	4.91 (1.58, 8.24)	**< 0.001**	1.79 (0.94, 2.64)	1.45 (0.90, 2.00)	**< 0.001**	3.21 (2.36, 4.05)
T1 - BL	612.5 (190, 1035)	**< 0.001**	1.85 (0.95, 2.74)	9.18 (5.86, 12.51)	**< 0.001**	3.34 (2.50, 4.19)	0.82 (0.27, 1.37)	**< 0.001**	1.80 (0.96, 2.65)
T2 - BL	561.1 (138, 984)	**0.003**	1.69 (0.80, 2.58)	8.73 (5.40, 12.05)	**< 0.001**	3.18 (2.33, 4.03)	0.82 (0.27, 1.37)	**< 0.001**	1.80 (0.96, 2.65)
T3 - BL	571.1 (148, 994)	**0.002**	1.72 (0.83, 2.61)	7.36 (4.04, 10.69)	**< 0.001**	2.68 (1.84, 3.53)	0.45 (-0.10, 1.00)	0.189	1.00 (0.15, 1.85)
T4 – BL	286.7 (-136, 709)	0.512	0.86 (-0.03, 1.76)	6.82 (3.49, 10.14)	**< 0.001**	2.48 (1.64, 3.33)	0.45 (-0.10, 1.00)	0.189	1.00 (0.15, 1.85)
T5 - BL	119.6 (-303, 542)	1.000	0.36 (-0.53, 1.25)	6.73 (3.40, 10.05)	**< 0.001**	2.45 (1.60, 3.30)	0.27 (-0.28, 0.82)	1.00	0.60 (-0.25, 1.45)
T6 - BL	76.3 (-346, 499)	1.000	0.23 (-0.66, 1.12)	7.64 (4.31, 10.96)	**< 0.001**	2.78 (1.93, 3.62)	0.18 (-0.37, 0.73)	1.00	0.40 (-0.45, 1.25)
T7 - BL	108.9 (-314, 532)	1.000	0.33 (-0.57, 1.22)	7.27 (3.95, 10.60)	**< 0.001**	2.65 (1.80, 3.50)	0.00 (-0.55, 0.55)	1.00	0.00 (-0.85, 0.85)
T8 - BL	64.0 (-359, 487)	1.000	0.19 (-0.70, 1.09)	7.36 (4.04, 10.69)	**< 0.001**	2.68 (1.84, 3.53)	0.09 (-0.64, 0.46)	1.00	-0.20 (-1.05, 0.65)

*Adjusted for age and lower body fat; adjusted for multiple comparisons of marginal means using Bonferroni's method; values marked in bold indicate statistically significant results ($p < 0.05$).
