# Supplementary material for: Acute Effects of Percussive Therapy on Thigh Muscle Microcirculation and Oxygenation
Source: J Funct Morphol Kinesiol. 2026 Apr 14;11(2):154. doi: 10.3390/jfmk11020154 (PMC13108165; doi:10.3390/jfmk11020154)
Supplement: Supplementary file 1 [file jfmk-11-00154-s001.zip › Table S4.pdf]

Table S4. Results of sensitivity analysis. Estimated mean differences from baseline for muscle microcirculation (MM) including participants with non-physiological signal artefacts (N=21)

Comparison	Estimate (95%CI)	P-value	Cohen's d
**T0 - BL**	270.1 (-851.2, 1391.4)	1.000	0.209
**T1 - BL**	972.9 (-148.4, 2094.2)	0.142	0.751
**T2 - BL**	928.8 (-192, 2050.1)	0.191	0.718
**T3 - BL**	679.6 (-441.7, 1800.9)	0.816	0.525
**T4 - BL**	607.8 (-513.5, 1729.1)	1.000	0.470
**T5 - BL**	350.5 (-770.8, 1471.8)	1.000	0.271
**T6 - BL**	816.3 (-770.8, 1471.8)	0.382	0.631
**T7 - BL**	275.4 (-845.9, 1396.7)	1.000	0.213
**T8 - BL**	815.6 (-305.6, 1936.9)	0.384	0.630

*Analysis based on N=21, including three participants excluded from the primary analysis due to non-physiological signal artefacts in post-intervention recordings. An identical linear mixed model was applied, adjusted for lower body fat percentage, age, and intervention duration. Bonferroni correction was applied for multiple comparisons. The overall time effect was not significant ($F(9,180) = 1.36$, $p = 0.210$). The direction of all effects was consistent with the primary analysis (N=18); however, statistical significance was not maintained due to substantially increased residual variance (residual SD = 1295 vs 313 in the primary analysis), attributable to the artefact-affected recordings. CI = confidence interval; BL = baseline.
